# A Narrative Review of Point of Care Ultrasound Assessment of the Optic Nerve in Emergency Medicine

**DOI:** 10.3390/life13020531

**Published:** 2023-02-15

**Authors:** Torey Lau, Justin S. Ahn, Rahim Manji, Daniel J. Kim

**Affiliations:** 1 Department of Emergency Medicine, University of British Columbia, Vancouver, BC V5Z 1M9, Canada; 2 Department of Emergency Medicine, St. Paul’s Hospital, Vancouver, BC V6Z 1Y6, Canada; 3 Department of Emergency Medicine, Royal Columbian Hospital, New Westminster, BC V3L 3W7, Canada; 4 Department of Emergency Medicine, Vancouver General Hospital, Vancouver, BC V5Z 1M9, Canada

**Keywords:** ultrasound, point of care ultrasound (POCUS), optic nerve, optic nerve sheath diameter (ONSD), intracranial pressure, idiopathic intracranial hypertension, optic neuritis, acute mountain sickness

## Abstract

Point of care ultrasound (POCUS) of the optic nerve is easy to learn and has great diagnostic potential. Within emergency medicine, research has primarily focused on its use for the assessment of increased intracranial pressure, but many other applications exist, though the literature is heterogeneous and largely observational. This narrative review describes the principles of POCUS of the optic nerve including anatomy and scanning technique, as well as a summary of its best studied clinical applications of relevance in emergency medicine: increased intracranial pressure, idiopathic intracranial hypertension, optic neuritis, acute mountain sickness, and pediatric intracranial pressure assessment. In many of these applications, sonographic optic nerve sheath diameter (ONSD) has moderately high sensitivity and specificity, but the supporting studies are heterogeneous. Further studies should focus on standardization of the measurement of ONSD, establishment of consistent diagnostic thresholds for elevated intracranial pressure, and automation of ONSD measurement.

## 1. Introduction

Point of care ultrasound (POCUS) is an essential diagnostic and evaluative tool in emergency medicine (EM). While applications such as focused assessment with sonography for trauma (FAST) and focused echocardiography are well established, other applications such as ultrasound assessment of the optic nerve may be less familiar to the average emergency physician (EP). Like other POCUS applications, the advantage of ultrasonography is the ability to perform rapid bedside assessment using a relatively inexpensive, portable, and non-invasive device. It does not require transportation of critically ill patients out of a monitored setting for conventional neuroimaging such as computed tomography (CT) and magnetic resonance imaging (MRI) [1,2]. Further, its use can evaluate the eye in challenging patient populations, such as in pediatrics or in the setting of trauma where swelling of the eyelid can interfere with conventional examination and direct ophthalmoscopy of the eye [3].

Ultrasonography of the optic nerve is best described in the literature for the evaluation of elevated intracranial pressure (ICP), but other applications exist. These include its use in idiopathic intracranial hypertension (IIH), optic neuritis, acute mountain sickness (AMS), and ventriculoperitoneal (VP) shunt assessment [2]. POCUS of the optic nerve may assist EPs in identifying critically ill patients, facilitating advanced imaging, or expediting transportation to appropriate referral centers if advanced neuroimaging capabilities are not available locally [2].

Through this review, we aim to describe the principles of POCUS of the optic nerve including anatomy and scanning technique and provide a narrative summation of its various clinical applications of relevance in EM.

## 2. Materials and Methods

A literature review was performed to identify publications relating to ocular POCUS assessment of the optic nerve in patients presenting to the emergency department (ED). The authors performed the search in October 2022 and searched PubMed, Embase, and Google Scholar from inception up to September 2022 using the keywords “emergency optic nerve ultrasound” and the MeSH terms “optic nerve/diagnostic imaging” combined with “ultrasonography”. The authors included retrospective and prospective studies, systematic reviews, meta-analyses, narrative reviews, clinical guidelines, and case series. Case reports, editorials, letters, and non-English language studies were excluded. Article inclusion was determined by author review and consensus based on clinical relevance to EM evaluation and management.

## 3. Results

A total of 43 articles were determined to be of relevance to EM clinicians and were included in this narrative review. Of these, 26 papers were quantitative clinical studies pertaining to clinical applications and are summarized in Table 1, Table 2, Table 3, Table 4 and Table 5.

## 4. Discussion

### 4.1. Anatomy and Physiology

The optic nerve carries approximately 1.2 million nerve fibers from retinal ganglion cells that join together at the optic disc before traveling through the optic chiasm towards the brain [30]. The nerve is surrounded by the pia mater, subarachnoid space, arachnoid mater, and dura mater, together forming the optic nerve sheath [31,32].

**Figure 1:**
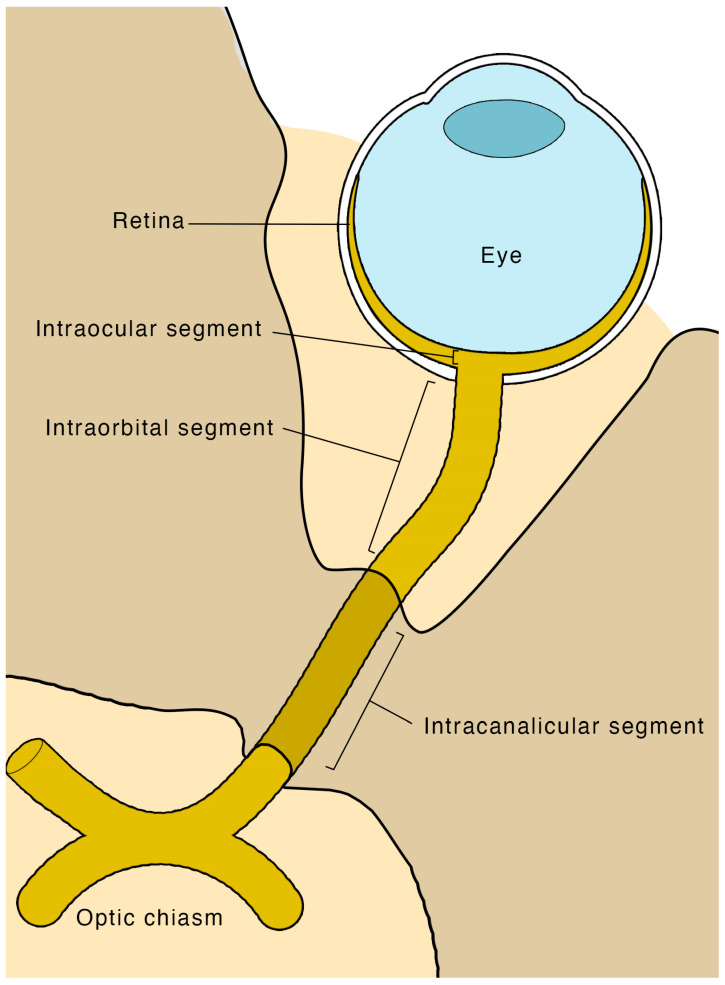
Schematic of the optic nerve and its different segments.

There are four segments to the optic nerve (Figure 1): the intraocular, intraorbital, intracanalicular, and intracranial segments. The intraocular segment is approximately 1 mm deep, 1.5 mm in diameter, and is clinically visible by direct ophthalmoscopy as the optic disc. Next, the intraorbital segment is 25–30 mm long and is the subject of POCUS of the optic nerve. It extends from the globe towards the optic foramen, measuring approximately 3–4 mm in diameter. The intracanalicular segment measures approximately 6 mm in length and travels through the optic canal. Ultimately the intracranial segment joins the optic chiasm [30].

The cranium is a fixed space containing the brain, blood, and cerebrospinal fluid (CSF) within the subarachnoid space, with little tolerance for increased volume. Significant increases in volume of any of the cranial components will lead to increased ICP [33]. Etiologies that increase the volume of cranial contents include cerebral edema, tumors, intracranial hemorrhage, increased CSF production, and CSF outflow obstruction [33,34].

The subarachnoid space of the optic nerve is continuous with the brain, and CSF slowly percolates throughout the entire subarachnoid space [8,30]. Experimental studies demonstrate a linear relationship between ICP and the subarachnoid pressure of the optic nerve [35]. Since the optic nerve sheath is distensible, it fluctuates in size based on changes in ICP [35,36]. This has been confirmed with in vivo studies demonstrating increases in sonographic optic nerve sheath diameter (ONSD) after intrathecal infusions, and normalization of ONSD after CSF decompression [37].

### 4.2. Indications and Contraindications

EPs working in a busy department will most effectively use POCUS to answer specific clinical questions to guide further management of patients. The primary question is whether there is sonographic evidence of elevated ICP. As such, clinical indications generally revolve around the suspicion of raised ICP, including presentations of head trauma, headache, stroke, or altered level of consciousness [2]. In trauma patients with periorbital swelling, POCUS allows ocular assessment for elevated ICP without requiring eyelid retractors or sedation to examine for papilledema. Furthermore, POCUS of the optic nerve may be useful for assessing and monitoring ICP in patients who are sedated and paralyzed, such as the intubated patient, where neurological examination is unreliable. Other indications relate to the inability to access advanced neuroimaging or lack of specialist support, such as in rural or remote settings, or in patients that are too unstable to leave a monitored setting for advanced imaging [38].

The main contraindication to POCUS of the eye and optic nerve is suspected or confirmed globe rupture, but this may not be apparent to the clinician due to periorbital swelling or hematoma. Therefore, it is imperative to use plenty of ultrasound gel and to avoid direct ocular pressure while scanning in the setting of trauma [39]. Another relative contraindication is uncontrolled open angle or acute angle glaucoma [2].

### 4.3. Scanning Technique and Sonographic Anatomy

**Figure 2:**
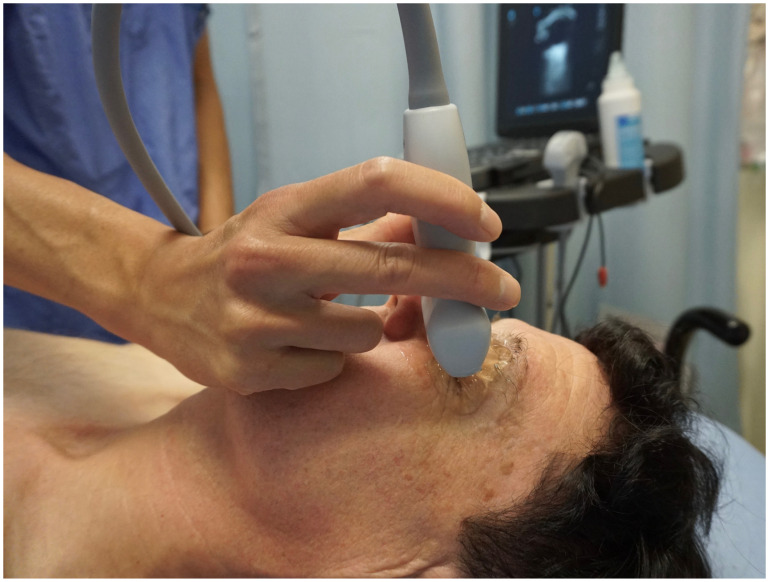
Demonstration of point of care ultrasound of the eye. Plenty of ultrasound gel should be used and the hand can be steadied over the patient’s zygomatic process to minimize downward pressure on the eye applied by the probe. A clear, protectant film may be used to protect the eye if desired. This image was obtained with consent from the model for publication.

POCUS of the optic nerve is performed using a high-frequency linear transducer (5–14 MHz) on an ultrasound machine, with a smaller probe footprint providing better sonographic contact with the eye [33,40]. An ocular preset should be used, limiting thermal index to ≤1 and mechanical index to ≤0.23 in accordance with the as low as reasonably achievable (ALARA) principle [39,41]. Patients are placed in a comfortable position, generally supine, and asked to close their eyes with their head in a neutral position (Figure 2) [42]. Copious sterile ultrasound gel should be placed on the closed eyelids of the patient, and the probe should be placed gently on top of the gel to avoid direct pressure on the eye. The sonographer’s hand can be steadied over the patient’s zygomatic process or nasal bone to regulate the amount of downward pressure applied by the probe. If preferred, a transparent protective film may be placed over the eye to protect it, but care must be taken to avoid air entrapment between the film and external eyelids, as this will degrade image quality [41]. Scanning should be performed using B-mode in the transverse orientation. The patient should be asked to look forward at a fixed point in neutral gaze to minimize eye movement [2].

The optic nerve can be identified as a homogeneous hypoechoic linear structure extending far-field from the anechoic ocular globe [41]. It is surrounded by a more hyperechoic band representing the optic nerve sheath, analogous to the arachnoid membrane surrounding the subarachnoid space, which is further enclosed by a hypoechoic outer band representing the dura mater [2,43]. Finally, hyperechoic retrobulbar fat surrounds the optic nerve. The central retinal artery and vein run through the middle of the optic nerve, while the ophthalmic artery runs parallel adjacent to the optic nerve [44].

**Figure 3:**
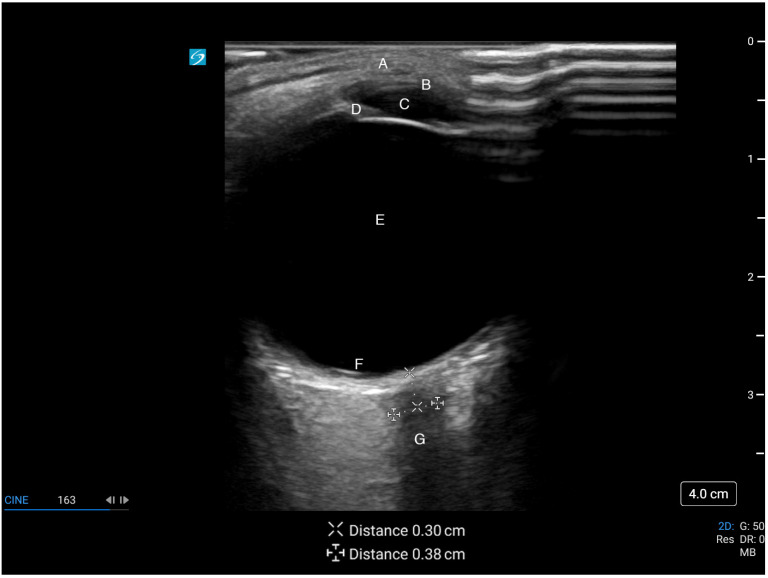
Ultrasound image of the eye and optic nerve. A: eyelid, B: cornea, C: anterior chamber, D: iris, E: vitreous chamber, F: retina, G: optic nerve with optic nerve sheath diameter of 3.8 mm, measured at 3 mm from the retina. This figure was obtained with consent from the model for publication.

Traditionally, ONSD is measured 3 mm behind the globe where the sheath is the most distensible and its measurements are reproducible [36,40]. Though there is recent evidence suggesting no difference in ONSD when measured between 3 and 5 mm posterior to the globe [45], most studies base their measurements at 3 mm [43]. The ONSD is measured between the outer hyperechoic borders of the subarachnoid space (Figure 3) [2,46]. If no hyperechoic stripe is visualized, the diameter is measured at the border of the hypoechoic optic nerve and the hyperechoic retrobulbar fat [32]. The measured diameter should be perpendicular to the longitudinal axis of the optic nerve [43]. Color doppler may aid visualization of a tortuous optic nerve by visualizing the midline central retinal artery and vein, allowing for accurate measurement of the ONSD perpendicular to the true course of the optic nerve [44,47]. However, the use of doppler must be minimized to avoid ocular injury [39]. Averaging 2–3 measurements in transverse or sagittal planes may minimize measurement errors [40,41]. Like other ultrasound applications, repeated measurements as clinically indicated may provide additional dynamic information [2].

POCUS of the optic nerve can easily be learned by novice users with minimal ultrasound experience after a brief training session [48]. EPs can accurately measure ONSD with ultrasound when compared to ONSD measurements on CT by a radiologist [49], and generally have good inter-rater reliability regardless of level of ultrasound training, though some variations exist amongst residency-level physicians [38,50].

### 4.4. Clinical Applications: Increased Intracranial Pressure

The most prominent clinical application of optic nerve POCUS is its diagnostic ability to detect elevated ICP. Kim et al. conducted a prospective observational study in 2021, comparing ultrasound assessment of ONSD with CT of the brain performed within 30 min in patients suspected of raised ICP in the ED [4]. From a total of 199 enrolled patients, 57 were found to have signs of raised ICP on CT scan. The median ONSD on ultrasound in the raised ICP group was significantly higher compared with the normal ICP group (5.7 mm vs. 4.3 mm, *p* < 0.001). These results confirm the findings of another prospective observational study from 2019 by Hanafi et al. where 62 trauma patients were compared with 50 healthy controls [5]. Of the 55 trauma patients with increased ICP on CT scan, sonographic ONSD was 6.06 mm in these patients compared to 4.02 mm in healthy controls.

Ohle et al. published a systematic review and meta-analysis in 2015 comparing trials assessing sonographic ONSD to diagnose elevated ICP compared to CT as the reference standard [6]. They included 12 studies with 478 study subjects in ED and ICU settings and found that ONSD had a sensitivity of 96% (95% CI, 88–99%), specificity 92% (95% CI, 78–98%), diagnostic odds ratio (DOR) 319 (95% CI 79–1290), positive likelihood ratio (+LR) 12.5 (95% CI 4.2–37.5), and negative likelihood ratio (–LR) 0.05 (95% CI 0.02–0.14). The major limitation of this meta-analysis was the moderate-to-high heterogeneity of the included studies.

Recognizing that invasive monitoring is more accurate than CT to detect raised ICP, Robba et al. performed a meta-analysis in 2018 that only included studies using invasive ICP measurement (intraparenchymal, intraventricular, or lumbar puncture [LP]) as the reference standard [7]. Raised ICP was defined as >20 mmHg or >25 cmH_2_O. They included seven studies with a total of 320 patients and found a pooled DOR of 68 (95% CI 29–135), +LR 5.4 (95% CI 3.8–7.5) and −LR 0.09 (95% CI 0.05–0.15). Their results are more modest compared to Ohle’s systematic review, but this likely relates to a more robust reference standard, fewer included studies, and lower heterogeneity. An updated meta-analysis by Aletreby et al. in 2022 assessed ONSD compared to invasive ICP measurement as the reference standard [8]. They included nine additional studies compared to Robba’s systematic review for a total of 619 patients. Their results were similar: pooled sensitivity of 90% (95% CI 85–94%), specificity 85% (95% CI 80–89%), DOR 47 (95% CI 26–83), +LR 6.1 (95% CI 4.4–8.5), and −LR 0.11 (95% CI 0.07–0.18).

The largest meta-analysis of optic nerve POCUS was published by Koziarz et al. in 2019 and included trials with participants in all age groups, sonographers of any training level, and used any reference standard as the comparator [9]. Their analysis included 71 studies with a total of 4551 patients and found a sensitivity and specificity in the traumatic brain injury subgroup of 97% (95% CI 92–99%) and 86% (95% CI 74–93%), respectively. In non-traumatic brain injury, sensitivity was mildly reduced at 92% (95% CI 86–96%) while specificity was similar at 86% (95% CI 77–92%).

Most studies used differing ONSD thresholds to diagnose increased ICP [4,8]. In 2019, Kim et al. attempted to establish a single sonographic ONSD cut point to detect elevated ICP [10]. They included six studies with 352 participants in their final analysis, all utilizing 5 mm as a cut point. They found this cut point provided a pooled sensitivity of 99% (95% CI 96–100%), specificity 73% (95% CI 65–80%), DOR 178 (95% CI 53–599), +LR 4.6 (95% CI 2.0–10.9), and −LR 0.05 (95% CI 0.02–0.14).

In comparison, the physical exam is generally poorly sensitive and specific for elevated ICP. Fernando et al. compared multiple modalities to diagnose elevated ICP, including physical exam and sonographic ONSD. Their meta-analysis found pupillary dilation was insensitive at 28% but moderately specific at 86%. Motor posturing had poor sensitivity and specificity at 54% and 64%, respectively. Decreased level of consciousness was more sensitive at 76% but less specific at 40% [11]. In comparison, sonographic ONSD measurement had a pooled area under the receiver operating characteristic (ROC) curve of 0.94. These authors did not calculate pooled sensitivity and specificity as the included studies used many different optimal cut points for elevated ICP. Finally, while direct ophthalmoscopy can detect elevated ICP based on the presence of papilledema, it is infrequently and poorly performed by EPs [51].

Based on the literature, optic nerve POCUS using an ONSD threshold of 5 mm appears to be a clinically useful and accurate tool to detect elevated ICP in the emergency setting. In general, it seems to be more sensitive than specific, which is clinically appropriate given that it would be undesirable to miss a case of high ICP. However, the lack of large randomized controlled studies limits the widespread use of this tool. POCUS assessment of ONSD cannot replace CT or invasive monitoring to diagnose elevated ICP, but it can be used in an appropriate clinical context to monitor at-risk patients, in patients too unstable to leave a monitored setting, or as a supplementary test in settings where access to advanced neuroimaging is limited.

### 4.5. Clinical Applications: Idiopathic Intracranial Hypertension

IIH is a neurologic disorder characterized by diffuse headache, visual abnormalities, and papilledema, most commonly affecting young females with increased body mass index (BMI) [16]. Physicians who are not ophthalmologists may have difficulty performing ophthalmoscopy for a variety of reasons, including limited equipment, lack of experience, or patient factors [52]. Therefore, POCUS of the optic nerve can be an effective alternative means to assess for elevated ICP and assist with the diagnosis of IIH.

Case-control studies have assessed the diagnostic accuracy of sonographic ONSD to detect elevated ICP to diagnose IIH, generally finding good sensitivity and specificity with varying diagnostic thresholds. Dağdelen et al. measured ONSD in 47 subjects with IIH and 50 healthy controls. They found the mean ONSD in IIH patients was increased at 6.4 mm compared with 4.9 mm in controls (*p* < 0.001). They identified an optimal cut point of 5.7 mm, yielding a sensitivity of 100% and specificity of 98% [12]. However, Kishk et al. found more modest results when they compared 99 females with both clinically definite and probable IIH to 35 healthy controls. All cases had both neurologic and ophthalmologic assessment including neuroimaging to rule out other diagnoses. ONSD measurement was performed prior to diagnostic LP. Mean sonographic ONSD was higher in cases than controls (6.57 mm vs. 5.50 mm, *p* < 0.001). Using a cut point of 6.05 mm, sonographic ONSD had only modest sensitivity but good specificity at 73% and 91%, respectively [13]. Their more modest results may be due to the inclusion of nine cases with probable IIH. Smaller studies have demonstrated good diagnostic utility for ONSD measurement. Del Saz-Saucedo et al. studied 30 subjects with suspected IIH, of which 19 were diagnosed with a positive LP. Using a higher cut point of 6.3 mm, sonographic ONSD had a sensitivity of 95% (95% CI 82–100%) and specificity of 91% (95% CI 69–100%) [14]. Finally, Ebraheim et al. studied 24 patients with suspected IIH, of which 20 were diagnosed with a positive LP. Sonographic ONSD had a sensitivity of 88% and specificity of 100% using a cut point of 6.2 mm [15].

Multiple studies have demonstrated dynamic changes in sonographic ONSD after LP. Jeub et al. found that removal of 30 mL of CSF by LP led to a reduction in ONSD by 0.4 mm and 0.5 mm in the right and left eyes, respectively [16]. In another study, Del Sez-Saucedo found that ONSD decreased by a mean of 0.9 mm after therapeutic LP achieved a CSF pressure of <15 cmH_2_O [14]. Even without therapeutic LP, Ebraheim found that after 4 weeks of treatment with acetazolamide alone, ONSD decreased by a mean of 0.4 mm [15].

ONSD appears to have good sensitivity and specificity to diagnose IIH. Further, it can be used to monitor the response to treatment. However, no standardized cut points currently exist, limiting its use as a diagnostic tool. Ultimately, more studies are required to establish a standard cut point to serve as a diagnostic threshold for IIH.

### 4.6. Clinical Applications: Optic Neuritis

Optic neuritis is an acute inflammatory disorder of the eye that causes vision disturbance or loss and ocular pain. It is most commonly idiopathic but may be secondary to other disease states, most notably multiple sclerosis [30]. Diagnosis is largely clinical and is aided by MRI [30,53]. Numerous case reports describe increased ONSD when measured by POCUS in patients with optic neuritis, leading to observational studies to research its potential as a diagnostic tool [54,55,56].

Lochner et al. studied 21 patients with first-episode demyelinating unilateral optic neuritis and 21 matched controls. All patients underwent MRI imaging to rule out other causes for their symptoms. They determined that sonographic ONSD was significantly increased compared to the unaffected eye, median 6.3 mm vs. 5.5 mm, respectively, and ONSD was larger in affected eyes than in controls. ONSD of the unaffected eye was similar to controls [17]. Kwon et al. studied 17 patients with new-onset unilateral optic neuritis and found median ONSD to be modestly higher in affected eyes than in unaffected eyes, measuring 5.51 mm and 5.05 mm, respectively [18]. While their findings are modest, they are supportive of the findings from Lochner’s study. As only two studies exist, more research is required to establish sonographic ONSD as a useful tool to diagnose optic neuritis.

### 4.7. Clinical Applications: Acute Mountain Sickness

Optic nerve sheath sonography has also been explored as a portable and non-invasive surrogate measure for increased ICP in acute altitude illnesses. Although causality is not clearly established, increased ICP has been reported in patients with AMS and high-altitude cerebral edema (HACE). Both entities often overlap with high altitude pulmonary edema (HAPE) [57,58].

Analysis of trekkers at high altitude has demonstrated positive correlation between ONSD and AMS. Fagenholz et al. studied travelers at 4240 m and found the mean ONSD in 69 patients with AMS was significantly larger than 218 patients without AMS. They also identified a positive correlation between ONSD and the Lake Louise Score (LLS) used to diagnose AMS [19]. Similarly, Sutherland et al. followed 13 mountaineers and took serial measurements from sea level to 6400 m, finding a positive correlation between increasing altitude, ONSD, and LLS [20].

However, analysis of healthy volunteers with non-exertional ascent to high altitude has shown conflicting results. Lawley et al. measured ONSD in 23 subjects at sea level and then at 3777 m via cable car ascent. They found ONSD increased significantly with altitude compared to baseline, but the increase was similar in those with and without symptoms of altitude illness [21]. Keyes et al. measured ONSD in a cohort of healthy patients at 1400 m, after rapid ascent to 4300 m via vehicle, and again after oxygen therapy. They found a small increase in mean ONSD in patients with AMS compared to patients without AMS [22]. However, the association was weak, and the majority of ONSD measurements were <5 mm, which is below the typical cut point for elevated ICP.

In contrast, Strapazzon et al. followed a cohort of 19 healthy volunteers and measured ONSD at baseline (262 m) and after helicopter ascent to 3830 m. They found that ONSD increased with altitude in all participants with a peak at 24 h and remained increased at 8 days [23]. In particular, the increase was more pronounced in patients who developed AMS, although this only comprised three participants. Kanaan et al. followed a cohort of 86 healthy adults who drove from 1240 m to 3545 m and then hiked up to 3810 m. All their subjects had an increase in ONSD with ascent that was not statistically significant, regardless of AMS diagnosis [24]. However, the degree of increase was higher in patients with AMS than patients without AMS. This suggests individual variability, so changes in an individual’s ONSD from baseline may be more important for the diagnosis of AMS compared to a standardized cut point.

The discrepant results are best summarized by Lochner’s systematic review. They included six studies with 436 patients but found considerable clinical and methodological heterogeneity across the studies that prevented them from performing a meta-analysis. The studies varied with respect to variables such as ascent time, confounding factors related to method of transport to altitude, interval measurements, and scanning protocol. With regards to ONSD, there was a significant degree of individual variability across the studies and within each study, limiting ONSD as a diagnostic tool for AMS [59].

Further research is necessary to clearly establish whether there is a correlation between ONSD and AMS. Ideally, there needs to be a large longitudinal cohort study measuring ONSD at baseline followed by a range of altitudes at consistent time intervals, using a standardized scanning protocol with assessment of interobserver reliability, and controlling for confounding factors such as medication use (such as acetazolamide and dexamethasone), ascent time, and acclimatization periods.

### 4.8. Clinical Applications: Pediatrics

There is particular interest in POCUS of the optic nerve in pediatric populations where radiation exposure for diagnostic imaging and invasive procedures are ideally avoided if reasonably possible. However, the measurement of ONSD in children is more complicated than in adults. ONSD increases with age, most rapidly in the first year of life [60]. Furthermore, ONSD may be affected by the patency of the anterior fontanelle and head circumference [27,41,61,62]. Because of these factors, no standardized ONSD normal value exists, though numerous diagnostic cut points for raised ICP have been suggested. One commonly cited ONSD cut point is 4 mm in children ≤1 year old, 4.5 mm in children 1 to 15 years old, and 5 mm in children >15 years old [41,60].

Bhargava et al. performed a systematic review and meta-analysis of 11 publications to investigate the performance of sonographic ONSD to detect increased ICP in a pediatric population. Their study included patients with acute or chronic causes of elevated ICP. When compared to any reference standard, they found a pooled sensitivity of 93% (95% CI 74–99%), specificity 74% (95% CI 52–88%), +LR 3.55 (95% CI 1.67–7.54), −LR 0.10 (95% CI 0.02–0.46), and DOR 39 (95% CI 4.16–365) with moderate-to-high heterogeneity when compared to any reference standard. When limited to an invasive reference standard, sensitivity was 89% (95% CI 63–97%), specificity 74% (95% CI 47–90%), +LR 3.36 (95% CI 1.36–8.31), −LR 0.15 (95% CI 0.04–0.68), and DOR 22 (95% CI 2–192) with minimal heterogeneity [25].

Şik et al. studied 147 children presenting to the ED with head trauma requiring a CT head. ONSD ultrasound was performed by a pediatric EM fellow who was blinded to clinical and radiologic findings. When compared to CT as the reference standard, they found a high sensitivity of 93% and specificity of 94% when using a cut point of 5.1 mm amongst all age groups [26]. However, others found sonographic ONSD to have lower diagnostic performance consistent with the results of Bhargava’s systematic review. Padayachy et al. performed a relatively large observational study comparing sonographic ONSD to invasive ICP measurements. They included 56 children ≤1 year old and 118 children >1 year of age. In children ≤1 year old using a cut point of 5.16 mm to detect ICP above 20 mmHg, sonographic ONSD had a sensitivity of 80% (95% CI 44–98%) and specificity of 76% (95% CI 61–87%), while in children >1 year, a cut point of 5.75 mm produced a sensitivity of 86% (95% CI 75–93%) and specificity of 70% (95% CI 56–82%). They found children with an open anterior fontanelle had the poorest correlation between sonographic ONSD measurements and the reference standard [27]. Ultimately, ONSD appears to have high sensitivity and only modest specificity to detect raised ICP in pediatric populations, but further research is required, especially to determine age-appropriate cut points.

There has been increasing interest in utilizing sonographic ONSD to detect VP shunt failure. While VP shunt malfunction results in raised ICP, children often present with non-specific symptoms overlapping with benign childhood ailments [63]. While imaging is heavily relied upon to diagnose VP shunt failure, CT and MRI are insensitive and children often require repeat CT imaging, thereby raising the risk of malignancy due to cumulative radiation exposure [64]. Lin et al. studied 32 patients with suspected VP shunt failure and compared sonographic ONSD measured by pediatric EPs against neuroimaging and a neurosurgical clinical diagnosis as reference standards. When compared to neuroimaging, sonographic ONSD had a sensitivity of 60% (95% CI 23–88%) and specificity of 67% (95% CI 48–81%). When compared to neurosurgical opinion, sensitivity rose to 75% (95% CI 30–95%) and specificity to 68% (95% CI 49–82%) [28]. Hall et al. studied 39 encounters suspicious for VP shunt failure and compared sonographic ONSD by pediatric EPs with neurosurgical diagnosis. They found sensitivity to be 61% (95% CI 36–83%) and specificity to be 22% (95% CI 6–48%) [29]. Unfortunately, sonographic ONSD appears to have poor diagnostic value in VP shunt failure, though larger studies in the future may provide clarity in this emerging area of interest.

### 4.9. Limitations and Future Directions

In general, clinical studies of sonographic ONSD are small and almost entirely observational. The literature is limited by heterogeneous patient populations, reference standards, and cut points. One contributing factor is the inconsistent reporting on how ONSD is measured which may contribute to varying diagnostic cut points [40]. Until larger and more methodologically rigorous studies are published, sonographic ONSD is more likely to play a complementary role, rather than replace advanced imaging or invasive monitoring. There may be a greater role for sonographic ONSD in rural EDs or austere environments where advanced imaging or invasive monitoring is unavailable or impractical. Establishing a widely accepted diagnostic threshold for raised ICP should be a priority before sonographic ONSD can be broadly adopted for clinical use. Automation and machine learning may be useful to standardize ONSD measurements, eliminating the risk of human measurement error in the future [65].

The pediatric literature is limited by the lack of established and broadly accepted diagnostic cut points for increased ICP. Future studies should clarify the specific factors affecting pediatric ONSD measurement and should identify widely accepted, standardized cut points based on age or other body measurements such as head circumference [25].

## 5. Conclusions

POCUS of the optic nerve appears to be a useful tool for several clinical applications, including traumatic brain injury, IIH, and optic neuritis. Though evidence for its use is largely observational, sonographic ONSD generally has high sensitivity and moderate specificity to diagnose conditions that have a pathophysiologic process resulting in elevated ICP. It may also detect evidence of optic nerve inflammation in acute optic neuritis. While POCUS of the optic nerve should generally complement traditional diagnostic algorithms and modalities until larger randomized trials exist, it may be an important decision-making tool in specific circumstances such as the unstable patient or in rural or remote settings. Future studies should prioritize standardizing ONSD measurement techniques and diagnostic cut points in both pediatric and adult populations.

## Figures and Tables

**Table 1:** Summary of quantitative clinical studies to diagnose increased intracranial pressure.

Author and Year	Study Design	Study Population	Intervention	Comparator	Results
Kim et al., 2021 [4]	Prospective observational study	199 adults with suspected raised ICP	Sonographic ONSD	CT scan	Median sonographic ONSD wider in raised ICP patients (5.7 mm vs. 4.3 mm)
					Ideal cut point of 5.3 mm Sensitivity 75% Specificity 91%
Hanafi et al., 2019 [5]	Prospective observational study	112 adults with traumatic headache vs. controls	Sonographic ONSD	CT scan	Mean sonographic ONSD wider in patients (6.06 mm vs. 3.04 mm)
					Ideal cut point of 5.3 mm yielding: Sensitivity 96% Specificity 71%
Ohle et al., 2015 [6]	Meta-analysis	478 adults across 12 studies with suspected raised ICP	Sonographic ONSD	CT scan	Sensitivity 96% Specificity 92% +LR 12.5 −LR 0.05
Robba et al., 2018 [7]	Meta-analysis	320 adults across 7 studies with suspected raised ICP	Sonographic ONSD	Invasive ICP measurement	DOR 68 +LR 5.4 −LR 0.09
Aletreby et al., 2022 [8]	Meta-analysis	619 adults across 16 studies with suspected raised ICP	Sonographic ONSD	Invasive ICP measurement	Sensitivity 90% Specificity 85% +LR 6.1 −LR 0.11 DOR 46.7
Koziarz et al., 2019 [9]	Meta-analysis	4551 patients of any age across 71 studies with suspected raised ICP	Sonographic ONSD	Any reference standard (CT or invasive ICP measurement)	Traumatic brain injury: Sensitivity 97% Specificity 86% +LR 6.9 −LR 0.04
					Non-traumatic injury Sensitivity 92% Specificity 86% +LR 6.9 −LR 0.09
					Optimal cut point of 5.0 mm
Kim et al., 2019 [10]	Meta-analysis	352 adults across 6 studies with suspected raised ICP	Sonographic ONSD with a cut point of 5.0 mm in all included studies	CT scan	Sensitivity 99% Specificity 73% +LR 4.6 −LR 0.05 DOR 178
Fernando et al., 2019 [11]	Meta-analysis	5123 adults across 40 studies with suspected raised ICP	Physical exam, sonographic ONSD, or CT imaging	Invasive ICP measurement or craniotomy with operative diagnosis of raised ICP	Pupillary dilation: Sensitivity 28% Specificity 86%
					Motor posturing: Sensitivity 54% Specificity 64%
					Decreased level of consciousness: Sensitivity 76% Specificity 40%
					Sonographic ONSD: AUROC 0.94
					CT absence or compression of basal cisterns: Sensitivity 86% Specificity 61%
					CT any midline shift: Sensitivity 81% Specificity 43%
					CT severe midline shift: Sensitivity 21% Specificity 89%

**Table 2:** Summary of quantitative clinical studies to diagnose idiopathic intracranial hypertension.

Author and Year	Study Design	Study Population	Intervention	Comparator	Results
Dağdelen et al., 2022 [12]	Case-control study	47 adults with IIH	Sonographic ONSD	50 healthy controls	Mean sonographic ONSD wider in cases (6.4 mm vs. 4.9 mm)
					Ideal cut point of 5.7 mm yielding: Sensitivity 100% Specificity 98%
Kishk et al., 2018 [13]	Case-control study	99 females with IIH (90 definite IIH, 9 probable IIH)	Sonographic ONSD	35 age-matched healthy controls	Mean sonographic ONSD wider in cases (6.57 mm vs. 5.50 mm)
					Ideal cut point of 6.05 mm yielding: Sensitivity 73% Specificity 91%
del Saz-Saucedo et al., 2016 [14]	Prospective observational	30 patients with suspected IIH	Sonographic ONSD	Lumbar puncture with CSF opening pressure of ≥25 cmH_2_O	Mean sonographic ONSD wider in IIH patients (6.8 mm vs. 5.7 mm)
					Idea cut point of 6.3 mm yielding: Sensitivity 95% Specificity 91% +LR 10.4 −LR 0.06
Ebraheim et al., 2018 [15]	Case-control study	24 adults with IIH (20 definite IIH, 4 probable IIH). All cases received acetazolamide treatment.	Sonographic ONSD	30 controls	Mean sonographic ONSD higher in IIH patients (6.7 mm vs. 5.5 mm)
					ONSD was not correlated with CSF opening pressure
					1 week post-LP (10–15 mL withdrawn), mean ONSD not significantly different from baseline (6.6 mm vs. 6.7 mm)
					4 weeks post-LP and after treatment with acetazolamide, mean ONSD decreased from baseline (6.4 mm vs. 6.8 mm)
					Ideal cut point of 6.2 mm yielding: Sensitivity 88% Specificity 100%
Jeub et al., 2020 [16]	Case-control study	19 adults with IIH (15 definite IIH, 4 probable IIH)	Sonographic ONSD	20 healthy controls	Mean sonographic ONSD higher in IIH patients (values not provided)
					24 h after LP (30 mL withdrawn), mean ONSD decreased (mean reduction right eye 0.4 mm, left eye 0.5 mm)
					Ideal cut point of 5.8 mm yielding: Sensitivity 81% Specificity 80%

**Table 3:** Summary of quantitative clinical studies to diagnose optic neuritis.

Author and Year	Study Design	Study Population	Intervention	Comparator	Results
Lochner et al., 2014 [17]	Case-control study	21 adults with unilateral first episode optic neuritis	Sonographic ONSD	Unaffected eye and 21 healthy age- and sex-matched controls	Median ONSD higher in affected eye than the unaffected eye (6.3 mm vs. 5.5 mm) and controls (6.3 mm vs. 5.2 mm)
Kwon et al., 2019 [18]	Prospective observational study	17 adults with unilateral first episode optic neuritis	Sonographic ONSD	Unaffected eye	Median ONSD higher in affected eye than in unaffected eye (5.5 mm vs. 5.0 mm)

**Table 4:** Summary of quantitative clinical studies to diagnose acute mountain sickness.

Author and Year	Study Design	Study Population	Intervention	Comparator	Results
Fagenholz et al., 2009 [19]	Prospective observational study	69 adults with AMS traveling through Pheriche, Nepal (4240 m)	Sonographic ONSD	217 adults without AMS	Mean ONSD higher in patients with AMS (5.3 mm vs. 4.5 mm)
					ONSD positively correlated with total LLS
					ONSD associated with AMS (OR 6.3)
Sutherland et al., 2008 [20]	Prospective cohort study	13 adult mountaineers ascending Mount Everest	Sonographic ONSD	N/A	An increase in 1000 m is associated with ONSD increase of 0.1 mm
					ONSD positively correlated with LLS
Lawley et al., 2012 [21]	Prospective cohort study	23 adults at sea level passively transported to 3777 m via cable car	Sonographic ONSD	N/A	Mean ONSD higher in patients at high-altitude than at sea level (5.6 mm vs. 5.2 mm)
					ONSD did not change when headache resolved with acclimatization
Keyes et al., 2013 [22]	Prospective cohort study	57 adults at 1400 m passively transported to 4300 m via car	Sonographic ONSD	N/A	Mean ONSD higher in AMS patients at altitude when compared with their baseline altitude (4.3 mm vs. 4.0 mm).
					Mean ONSD decreased in AMS patients at altitude after oxygen therapy (4.3 mm vs. 4.1 mm)
					Mean ONSD higher in AMS patients at altitude compared with non-AMS patients (4.3 mm vs. 4.0 mm)
Strapazzon et al., 2014 [23]	Prospective cohort study	19 adults ascending from 262 m to 3830 m passively via helicopter	Sonographic ONSD	N/A	Mean ONSD higher at altitude than at baseline (6.4 mm vs. 5.5 mm). ONSD increased in a parabola, peaking at 24 h and remained elevated at 8 days.
					ONSD associated with diagnosis of AMS
Kanaan et al., 2015 [24]	Prospective cohort study	86 adults ascending from 1240 m to 3545 m passively via car, then hiked and slept at 3810 m	Sonographic ONSD	N/A	Mean ONSD higher at altitude than at baseline (6.1 mm vs. 5.6 mm). This increase was larger in subjects with AMS than those without (mean 0.57 mm vs. 0.21 mm increase)
					Change in ONSD associated with diagnosis of AMS (OR 1.99)

**Table 5:** Summary of quantitative clinical studies to diagnose increased ICP or VP shunt failure in pediatric populations.

Author and Year	Study Design	Study Population	Intervention	Comparator	Results
Bhargava et al., 2020 [25]	Meta-analysis	543 children aged <18 years across 11 studies with suspected raised ICP	Sonographic ONSD	CT, MRI, invasive ICP measurement, or operative diagnosis	With any reference standard: Sensitivity 93% Specificity 74% +LR 3.6 −LR 0.1 0DOR 39
					With an invasive reference standard: Sensitivity 89% Specificity 74% +LR 3.4 −LR 0.15 DOR 22
Şik et al., 2022 [26]	Prospective observational study	147 children aged 1 month to 18 years with suspected raised ICP after head trauma	Sonographic ONSD	CT scan	Subjects with abnormal CT scans: ONSD associated with reduced level of consciousness and elevated ICP on CT report Ideal cut point of 5.1 mm yielding: Sensitivity 93% Specificity 94%
					Subjects without elevated ICP and with a space occupying lesion on CT Ideal cut point of 4 mm yielding: Sensitivity 75% Specificity 63%
Padayachy et al., 2016 [27]	Prospective observational study	56 children aged <1 year and 118 children between 1 and 14 years undergoing invasive ICP measurement under general anesthesia	Sonographic ONSD	Invasive ICP measurement	Subjects <1 year old, ideal cut point of 5.16 mm yielding: Sensitivity 80% Specificity 76% PPV 42% NPV 95% DOR 12.7
					Subjects 1–14 years old, ideal cut point of 5.75 mm yielding: Sensitivity 86% Specificity 70% PPV 78% NPV 81% DOR 14.5
					Subjects with an open anterior fontanelle, ideal cut point of 5.16 mm yielding: Sensitivity 86% Specificity 75% PPV 50% NPV 95% DOR 18
Lin et al., 2019 [28]	Prospective observational study	32 patients aged <21 years with suspected VP shunt failure	Sonographic ONSD with pre-determined cut point of >4 mm in infants ≤1 year and 4.5 mm in children >1 year	CT, MRI, or neurosurgical impression of VP shunt failure	Compared to neuroimaging (CT/MRI): Sensitivity 60% Specificity 67% +LR 1.8 −LR 0.6 PPV 25% NPV 90%
					Compared to neurosurgical impression: Sensitivity 75% Specificity 68% +LR 2.3 −LR 0.37 PPV 25% NPV 95%
Hall et al., 2013 [29]	Prospective observational study	39 encounters with children aged 6 months to 18 years with suspected VP shunt failure	Sonographic ONSD with pre-determined cut points of >4 mm in infants ≤1 year and 4.5 mm in children >1 year	Neurosurgical decision to operate on VP shunt failure	Mean ONSD lower in those with VP shunt failure (4.5 mm vs. 5.0 mm)
					Sensitivity 61% Specificity 22% PPV 44% NPV 36%
